# Characterization of fillets and skins from two varieties of genetically improved farmed Nile tilapia (*Oreochromis niloticus*)

**DOI:** 10.1371/journal.pone.0314928

**Published:** 2025-02-21

**Authors:** Vinicius José Campos, Eliane Gasparino, José Wellington Rodrigues Lima Júnior, Angélica de Souza Khatlab, Marisa Silva Bastos, Thais Pacheco Santana, Fernanda Losi Alves Almeida, Maria Luiza Rodrigues de Souza, Marcos Antonio Matiucci, Gislaine Gonçalves Oliveira, Carlos Antonio Lopes de Oliveira, Ricardo Pereira Ribeiro, Carolina Schlotefeldt, Isis Regina Santos de Oliveira, Julia Gasparino de Oliveira, Ana Paula Del Vesco

**Affiliations:** 1 Department of Animal Science, Federal University of Sergipe, São Cristóvão, Sergipe, Brazil; 2 Department of Animal Science, State University of Maringá, Maringá, Paraná, Brazil; 3 Department of Morphological Sciences, State University of Maringá, Maringá, Paraná, Brazil; 4 Graduate Program in Food Science, State University of Maringá, Maringá, Paraná, Brazil; 5 Department of Pharmacy, State University of Maringá, Maringá, Paraná, Brazil; Benha University, EGYPT

## Abstract

Genetically improved farmed tilapia (GIFT) is a strain of Nile tilapia (*Oreochromis niloticus*) developed for improved production and commercial parameters. Skin color, one of the characteristics distinguishing tilapia varieties, is an important phenotypic trait associated with qualitative and productive performance. This study aimed to assess fillet and skin characteristics in GIFT Nile tilapia with black and red skin phenotypes. For this, 24 GIFT Nile tilapia from the same spawning stock were divided into two groups based on skin color, namely a black variety (dark skin) and a red variety (reddish skin). There were no significant differences in biometric parameters between varieties. Fish of the red variety had higher 2 h post-slaughter pH and muscle antioxidant capacity and lower yellow-blue axis value (b*), luminosity (L*), thawing loss, cooking loss, and conjugated diene content (*P* < 0.05). The skin of black tilapia exhibited higher force in the progressive tear test tear, and higher strength, tensile strength, deformation, and elongation in the traction and stretching test than that of red tilapia. Black tilapia skin had higher collagen and hydroxyproline contents. The skin of the red variety exhibited higher expression levels of alpha 1, 2, and 3 type I collagen genes, lower expression levels of the vimentin gene, and higher antioxidant capacity. The results suggest that skin color phenotype may be linked to important metabolic pathways influencing fish fillet and skin traits. These findings can support future research aimed at identifying optimal varieties of Nile tilapia for specific purposes and optimizing the utilization of filleting waste.

## Introduction

Fish farming represents the primary economic activity of many communities around the world, serving as an accessible source of protein with high biological value for human nutrition. According to data from the United Nations’ Food and Agriculture Organization [1], the per capita consumption of fish increased significantly between 1970 and 2019, rising from 11.4 to 20.5 kg. Nile tilapia (*Oreochromis niloticus*) is a particularly prominent species of cultivated fish due to its tolerance to high stocking densities and water salinity, good adaptability to rearing environments, and excellent disease resistance [2]. The global production of Nile tilapia reached approximately 6.5 million tonnes in 2022, with 860,355 tonnes produced in Brazil alone [3].

Recent trends suggest that the consumption of tilapia meat is increasing, attributed to its high protein quality, affordable price, and easy preparation [4]. Nuryanto et al. [5] showed that Nile tilapia meat (boneless and headless fillet) had an energy value of 1 kcal/g, with protein being the most abundant macronutrient (18.46 g/100 g). The concentration of amino acids found by the authors was 21.56%, and the major amino acids were glutamic acid (3.45%), aspartic acid (2.16%), arginine (1.88%), lysine (1.84%), and leucine (1.69%). Additionally, tilapia meat contained 30.39% fat, comprising both saturated and unsaturated fatty acids, including oleic (8.13%), palmitic (7.87%), linoleic (3.67%), and stearic (4.30%) acids. Furthermore, tilapia meat contained high levels of calcium (74.58 mg/100 g) [5].

Fillets are the primary form used by the fishing industry to market tilapia. As this cut accounts for only 34.2% of the fish weight, filleting generates large amounts of waste, including carcass, viscera, and skin [6]. Designing utilization strategies for tilapia waste is important for minimizing environmental impacts, generating revenue, and adding value to the industrial process. Studies proposed various uses for tilapia waste, such as utilization in human food products [7,8], biofuel production [9], and biomedical applications [10,11]. Some investigations demonstrated the efficacy of Nile tilapia skin as a low-cost wound dressing for burn treatment [12,13].

Collagen fibers derived from aquatic organisms have attracted the attention of scientists from different fields. The reasons for this interest are many, including the low risk of disease transmission to humans, lack of religious restrictions, high cost-effectiveness, low molecular weight, good biocompatibility, and easy absorption by the human body [14]. Fish skin is an abundant source of gelatin and collagen, which can be hydrolyzed into bioactive peptides with antihypertensive, antioxidant, antimicrobial, neuroprotective, antihyperglycemic, and anti-aging properties. These properties are determined by the composition and amino acid sequence of bioactive peptides [15]. According to Reátegui-Pinedo et al. [16], external factors such as nutrition status and pathogen infection, as well as genetic factors, can change the configuration of amino acids in Nile tilapia skin.

Developed three decades ago for improved production and commercial performance, the genetically improved farmed tilapia (GIFT) strain is currently the most cultivated lineage of Nile tilapia worldwide [17]. In GIFT tilapia, skin color is an important phenotypic trait that can be used to distinguish different varieties [18] and is associated with various adaptive, reproductive, and productive characteristics [19]. In view of these considerations, this study aimed to biometrically characterize GIFT Nile tilapia with black and red skin phenotypes and determine the effect of skin color on fillet and skin characteristics. The findings are expected to contribute to the identification of optimal varieties of Nile tilapia for specific purposes and the appropriate utilization of filleting waste.

## Materials and methods

The experiment received approval from the Animal Research Ethics Committee (CEUA protocol No. 2942240423) at the State University of Maringá, Brazil.

### Fish specimens and body measurements

A total of 60 fingerlings of GIFT Nile tilapia (*O*. *niloticus*) were used in the study. Fish were divided into two experimental groups based on their skin color, which are referred to in this paper as the black variety (dark skin, *n* = 30) and the red variety (reddish skin, *n* = 30). The experiment lasted 150 days.

The fish were reared at the Experimental Fish Farming Station (CODAPAR, State University of Maringá, Maringá, Paraná, Brazil) in excavated tanks at a stocking density of 3 fish/m^3^. Water quality was maintained under standard conditions. Each tank was equipped with a conventional aeration system operated with on/off cycles of 30 min, resulting in 12 h of operation and 12 h of rest over a 24 h period. All fish received the same commercial feed, whose nutritional composition varied according to the rearing phase (Table 1). Feeding was carried out 8 times a day in the fingerling phase, 6 times a day in the pre-juvenile phase, 4 times a day in the juvenile and grower phases, and 2 times a day in the finisher phase.

**Table 1:** Crude protein and digestible energy contents of the commercial feed (Acqua Fish, SUPRA; São Leopoldo, RS, Brazil) fed to black and red Nile tilapia.

Rearing phase	Crude protein (%)	Digestible energy (kcal/kg)
Fingerling	56	3700
Pre-juvenile	50	3600
Juvenile	42	3600
Grower	36	3400
Finisher	32	3000

At the end of the 150-day experimental period, 12 black fish and 12 red fish with similar body weights were captured from the pond and stunned by heat shock in one part ice to one part water [20]. Subsequently, the fish were euthanized by severing the spinal cord [21]. The fishes were weighed and measured for total length, standard length, head length, back height, and back width.

After biometric assessment, the fish were eviscerated, and the skin on both sides was removed using specialized pliers. The procedure consisted of peeling the skin from the dorsal region near the head toward the caudal region. The samples were identified and weighed. Then, fragments of skin (*n* = 12 fish per variety) were excised from the upper-right part of the body, washed with ice-cold saline, placed in cryogenic tubes, and frozen in liquid nitrogen. Subsequently, skin samples were stored in a freezer at −80°C until analysis of gene expression and antioxidant capacity. For histological quantification of collagen types, skin fragments (without scales) measuring about 5 cm were collected from the upper-right side of the body from 7 individuals per treatment. These samples were washed thoroughly with cold sterile saline solution and then placed in microtubes containing 10% buffered formaldehyde solution for fixation. After samples were collected for gene expression and histological analyses, the remaining portions of skin (right and left sides) were placed in plastic bags and stored in a −20°C freezer. These samples were used for analysis of skin resistance parameters, hydroxyproline content, and collagen content.

The skinless fish were decapitated and their heads were weighed. Fillets were separated from the right and left sides of the body (without skin), weighed, and subjected to analysis of meat quality indicators. For analysis of antioxidant capacity, oxidative metabolites, and gene expression, muscle samples (*n* = 12 fish per variety) were collected from the upper-right side of the body, placed in cryogenic tubes, and frozen in liquid nitrogen. Subsequently, muscle samples were stored in a freezer at −80°C until analysis. Fish carcasses were weighed after filleting.

### Indicators of fillet quality

The meat quality indicators assessed in this study were pH, instrumental color, water activity (*a*_w_), thawing loss (ThL), cooking loss (CL), and texture profile. For these analyses, each fish was considered an experimental unit (*n* = 12 per fish variety).

pH, color, and *a*_w_ analyses were performed on refrigerated fillets collected from the left side of the body at 2 h post-slaughter. For ThL and CL determination, fillets were collected from the right side of the body and stored in a freezer at −20°C until analysis.

Fillet pH was measured 2 h after slaughter [21] and 12 h after thawing [22] using a digital pH meter (HI 99163, Hanna^®^ Instruments Brasil Ltda). The electrode was inserted into three different points of the fillet (dorsal, ventral, and central regions).

Color parameters were measured after slaughter at six different points on the ventral side of fillets by using a CR-400 colorimeter (Konica Minolta Sensing Americas, Inc.) at a 90° angle and room temperature [23]. Luminosity (L*), red-green axis (a*), and yellow-blue axis (b*) are expressed in the CIELAB color space [23]. Chroma and hue angle were calculated according to Gomes et al. [24].

Water activity was determined on samples at room temperature using an AquaLab 4 TE analyzer (Meter Group, USA).

For ThL assessment, frozen fillets (time 0) were weighed and thawed in a digital biochemical oxygen demand incubator (model SSBOD 342L) at 4°C. After 12 h, the fillets were weighed, and ThL (%) was calculated as follows: ThL = 100 × (Weight_time0_ –Weight_time12h_)/(Weight_time0_) [22].

Thawed fillets were placed on trays and left to stand on the bench for 30 min until they reached room temperature. The fillets were then weighed to obtain their initial weight and roasted on a grill (Cadence) lined with aluminum foil until their internal temperature reached 75°C [23]. No seasonings were added during cooking. The internal temperature of fillets was measured at three different points using a digital culinary thermometer (TP101, CITEX, Arapongas, Paraná, Brazil). After the desired temperature was reached, the fillets were removed from the grill, placed on trays on the bench for 5 min at room temperature, and weighed to determine the final weight. CL (%) was calculated as follows: CL = 100 × (Initial weight − Final weight)/Initial weight [25].

The texture profile of fillets was evaluated by determining hardness, elasticity, cohesiveness, adhesiveness, and chewiness according to Nascimento et al. [26].

### Antioxidant capacity and oxidative metabolites in muscle tissues

Antioxidant capacity was investigated by measuring 2,2-diphenyl-1-picrylhydrazyl radical (DPPH^•^) scavenging activity and 2,2′-azino-bis(3-ethylbenzothiazoline-6-sulfonate) radical (ABTS^•+^) reducing activity. Extracts were prepared by mixing 100 mg of muscle sample (*n* = 7 fish per variety) with 1000 μL of methanol. Samples were ground until completely dissociated by using a Fisher Scientific 150 portable homogenizer and centrifuged at 10,000 × *g* for 10 min at 4°C. The supernatants were used as crude extracts.

DPPH^•^ scavenging activity was analyzed as described by Brand-Wiliams et al. [27], with some modifications. Briefly, 400 μL of crude extract was added to a microtube containing 1600 μL of 0.06 mM DPPH^•^ solution in methanol. The reactions were maintained in the dark for 30 min. After this period, samples were read using an Evolution 300 UV-Vis spectrophotometer (Thermo Fisher Scientific) at 515 nm. DPPH^•^ scavenging activity of muscle tissues was determined using the following equation: DPPH^•^ activity (%) = [1 − (Sample absorbance/DPPH^•^ absorbance)] × 100.

The ABTS^•+^ assay was performed as described by Rufino et al. [28], with some modifications. Initially, a solution was prepared by mixing 5 mL of 7 mM ABTS^•+^ with 88 μL of 140 mM potassium persulfate. The reaction system was maintained in the dark for 16 h at room temperature. After this period, 1000 μL of the solution was diluted in ethanol to an absorbance of 0.70 ± 0.05 at 734 nm. Then, 40 μL of crude extract was added to a microtube containing 1960 μL of diluted ABTS^•+^ solution. After 6 min, the absorbance was read at 734 nm using an Evolution 300 UV-Vis spectrophotometer (Thermo Fisher Scientific). ABTS^•+^ activity (%) was determined using the following equation: ABTS^•+^ activity (%) = [(ABTS^•+^ absorbance − Sample absorbance)/ABTS^•+^ absorbance] × 100.

The determination of primary (conjugated dienes) and secondary (thiobarbituric acid reactive substances) lipid peroxidation and muscle protein oxidation (carbonylated protein) was performed as described by Khatlab et al. [29] and Pontes et al. [30].

### Skin resistance: Progressive tear, tensile, and elongation tests

The skin (*n* = 12 fish per variety) collected from the left side of the body was used for resistance determination according to the methods described by Matiucci et al. [31].

### Antioxidant capacity (DPPH^•^ scavenging activity) of the skin

For assessment of DPPH^•^ scavenging activity in skin tissues, crude extracts were prepared as described for muscle tissues. Then, 100 μL of skin extract was added to a microtube containing 900 μL of 0.06 mM DPPH^•^ solution in methanol. The reactions were maintained in the dark for 30 min. After this period, the samples were read using an Evolution 300 UV-Vis spectrophotometer (Thermo Fisher Scientific^TM^) at 515 nm. The DPPH^•^ scavenging activity of the skin was calculated as described for muscle tissues.

### Hydroxyproline and collagen extraction and quantification

Thawed skins (*n* = 7 samples per variety) were washed with distilled water. Any residual scales and flesh were removed from the skins using a laboratory spoon. Samples were washed with neutral detergent and peracetic acid for 20 min. The water was changed twice. The washing procedure was performed gently to avoid damaging the samples. After cleaning, the skins were weighed and subjected to a pretreatment with acetic acid at 4.5% of the sample weight. Then, the skins were heated for 3.5 h. The liquid fraction containing hydroxyproline and collagen was filtered and left to cool. The extract was dried in a forced air oven for approximately 12 h, ground, and subjected to hydroxyproline and collagen quantification.

Hydroxyproline content was determined based on the oxidation of free hydroxyproline to pyrrole-2-carboxylic acid by chloramine-T, as described by Bergman and Loxley [32]. The absorbance was measured at 558 nm using a spectrophotometer. Hydroxyproline content was calculated from a standard curve. Then, collagen contents were calculated from hydroxyproline contents using a conversion factor of 7.25 (Collagen content = Hydroxyproline content/100 × 7.25).

### Determination of the area occupied by type I and type III collagen in the skin of black and red Nile tilapia

After fixation, the samples (*n* = 7 per variety) were dehydrated in an increasing series of ethanol, cleared in xylene, and embedded in paraffin for histological sectioning. Semi-serial sections measuring 3 μm in the longitudinal direction were obtained using a manual microtome (Leica Biosystems, RM2125 RTS).

**Fig 1:**
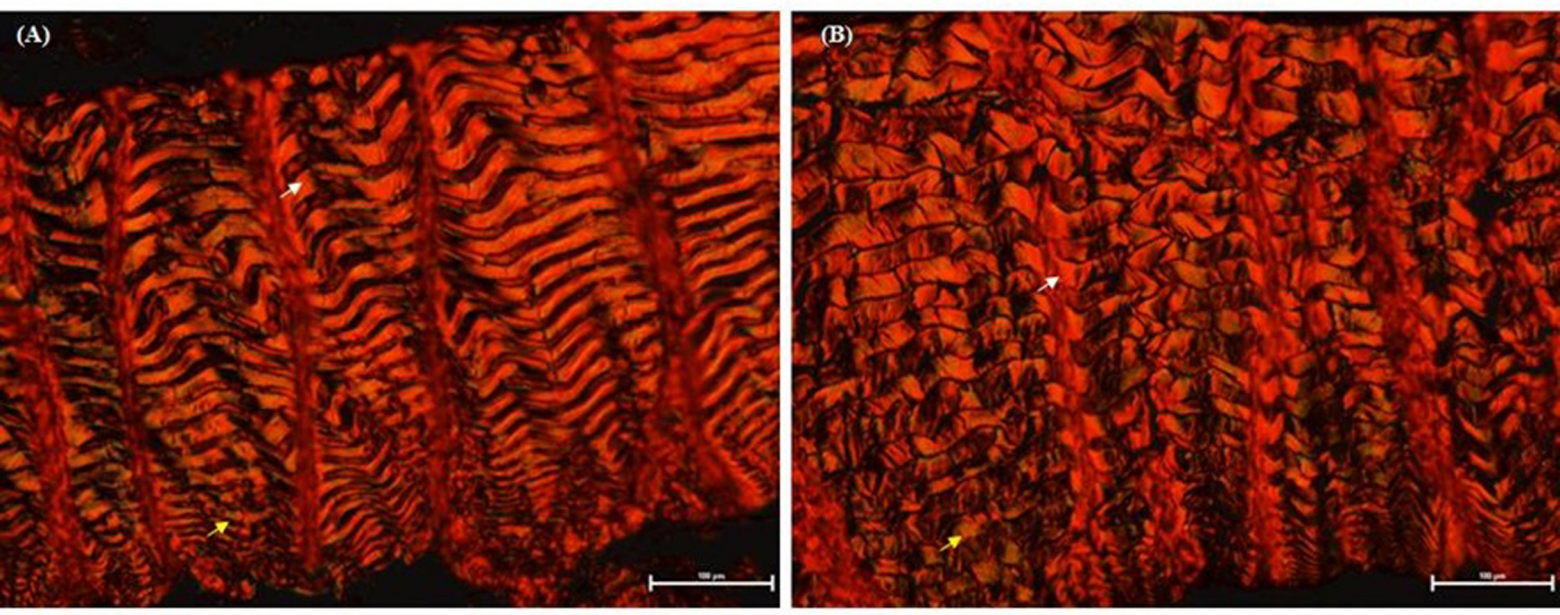
Histological sections of the skin of (A) black and (B) red Nile tilapia (*Oreochromis niloticus*) stained with Picrosirius Red and observed under a polarized light microscope (20× magnification). Type I collagen fibers are shown in red-orange (white arrows), and type III collagen fibers are shown in green (yellow arrows). Bar = 100 μm.

Histological sections were stained with Picrosirius Red to differentiate collagen types (Fig 1). For determination of the area occupied by type I and III collagen fibers, 10 images were captured per fish using a Nikon optical microscope (Eclipse^®^, Shinjuku, Japan) coupled to a high-resolution camera (Nikon^®^, DS-Fi1c, Shinjuku, Japan) with a 20× objective and optical polarization. Images were processed using NIS-Elements software (version 4.0, Prague, Czech Republic). Type I and III collagen fibers were quantified using Image-Pro Plus version 4.0 (Media Cybernetics). The results are expressed as a percentage, representing the relative area of each collagen type to the dermis area analyzed.

### Genes expression in skin and muscle tissues

The total RNA of skin and muscle samples (*n* = 7 per variety) was extracted using TRIzol reagent (Invitrogen, Carlsbad, CA, USA) according to the manufacturer’s instructions. The concentration of total RNA was measured using a NanoDrop 2000c spectrophotometer (Thermo Fisher Scientific) at a wavelength of 260 nm. The purity and integrity of total RNA in each sample were determined using the 260/280 nm and 260/230 nm absorbance ratios, which yielded values ranging from 1.9 to 2.1. Total RNA was treated with the DNase I amplification grade kit (Invitrogen, Carlsbad CA, USA) to avoid contamination with genomic DNA, according to the manufacturer’s instructions. Complementary DNA (cDNA) was synthesized using the SuperScript III First-Strand Synthesis Super Mix kit (Invitrogen Corporation), following the manufacturer’s instructions. cDNA samples were stored at −20°C until use. Real-time quantitative polymerase chain reactions (RT-qPCR) were conducted in duplicate using PowerUp^TM^ SYBR^TM^ Green Master Mix (Applied Biosystems, Vilnius, Lithuania) and a StepOne Real-Time PCR System version 2.3 (Applied Biosystems). The thermal cycling parameters for all genes followed the recommendations of the PowerUp SYBR^®^ Green Master Mix protocol (Applied Biosystems, Vilnius, Lithuania).

The genes alpha 1 type I collagen (*col1a1*, GenBank accession number AB603656.1), alpha 2 type I collagen (*col1a2*, GenBank accession number AB603657.1), alpha 3 type I collagen (*col1a3*, GenBank accession number AB603658.1), vimentin (*vim*, GenBank accession number XM_003438066.5), DEAH box helicase 9 (*dhx9*, GenBank accession number XM_025900168.1), ryanodine receptor 1 (*ryr1*, GenBank accession number XM_005474818.1), and ryanodine receptor 3 (*ryr3*, GenBank accession number XM_005453445.1) (Table 2) specific to *O*. *niloticus* were designed based on the sequences available on the NCBI website (www.ncbi.nlm.nih.gov), using the primer design software provided by IDT (www.idtdna.com). The beta-actin gene specific to *O*. *niloticus* was used as endogenous control (GenBank accession number EU887951.1) (Table 2).

**Table 2:** Gene sequences used in RT-qPCR reactions.

Gene	Sequence (5′→3′)	Amplicon size (bp)
*col1a1*	F: GAAGGGACACAGAGGATTCAC	148
	R: GCTCACACCATCCTTACCAG	
*col1a2*	F: GTGGATTCTACTGGATTGACCC	147
	R: TCTTGTTCTCGGTGCTTCTG	
*col1a3*	F: GCAGTGGATTTGAGTTCGTC	146
	R: CTTCTGTGTCAGGGTCTTAAGG	
*vim*	F: GACCATTGAGACCAGGGATG	136
	R: TGTAGCCCGAGTGAAATGTG	
*dhx9*	F: TTAACGGGTTCACGGAATAGG	113
	R: GGATGTCGTAGTTTGGAGTCAG	
*ryr1*	F: TTCTACCAACACCCCAATCTG	143
	R: AGTAACACAGGAAACGACAGC	
*ryr3*	F: TGTTTCATCTGTGGGATCGG	140
	R: GTGTGCTCTGTCTCATCCTTG	
β-actin	F: TGGTGGGTATGGGTCAGAAAG	217
	R: CTGTTGGCTTTGGGGTTCA	

bp, base pairs; *col1a1*, *collagen type I alpha 1* gene; F, forward; R, reverse; *col1a2*, collagen type I alpha 2 gene; *col1a3*, collagen type I alpha 3 gene; *vim*, vimentin; *dhx9*, DEAH box helicase 9 gene; *ryr1*, ryanodine receptor 1 gene; *ryr3*, ryanodine receptor *3* gene.

**Fig 2:**
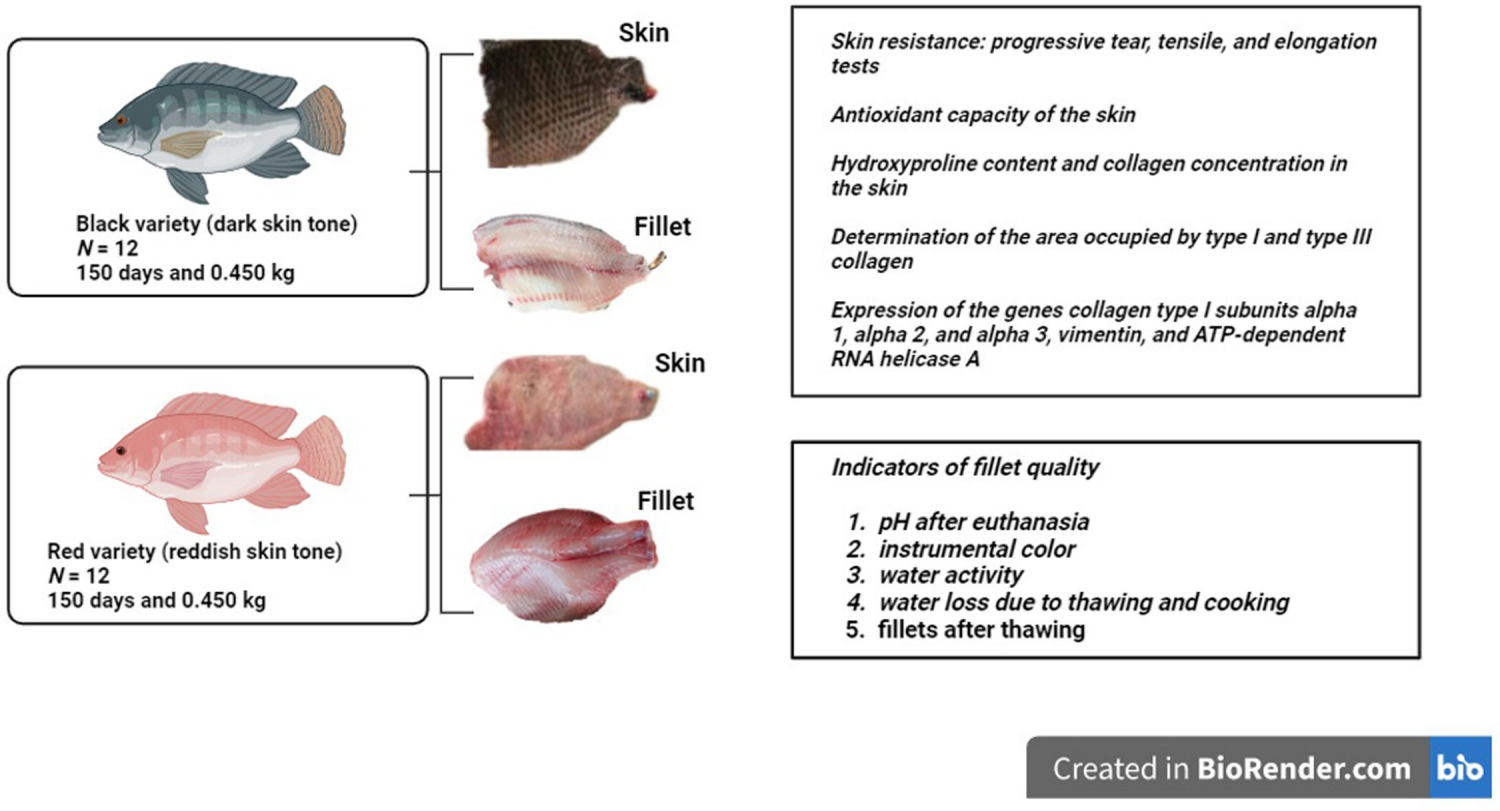
Experimental scheme and analyses.

The 2−^ΔCt^ method [33], where ΔCt = (Ct_targetgene_ − Ct_endogenousgene_), was used for the relative quantification of gene expression. The results are presented as arbitrary units (AU). The scheme of the experimental design is illustrated in Fig 2.

### Statistical analysis

The Shapiro–Wilk test was performed to assess the normality of the data. The data were analyzed using one-way ANOVA. The following statistical model was used: *Y*_*ij*_ = *μ* + *α*_*i*_ + *e*_*ij*_, where *Y*_*ij*_ is the dependent variable, *μ* is the general mean, *α*_*i*_ is the effect of treatment (*i* = black or red variety), and *e*_*ij*_ is the residual error. Means with significant differences were compared using Student’s *t*-test (*P* < 0.05) (SAS, 2002, version 9.00, SAS Inst. Inc., Cary, NC) [34].

## Results

No significant differences in biometric parameters were observed between tilapia varieties (Table 3).

**Table 3:** Body biometrics of black and red Nile tilapia (*Oreochromis niloticus*).

Trait	Variety		*P*-value
	Black	Red	
Body weight (g)	837.69 ± 186.69	857.33 ± 145.74	0.7733
Skin weight (g)	56.00 ± 14.22	52.08 ± 10.03	0.4379
Fillet weight (g)	266.38 ± 39.03	300.08 ± 42.50	0.0502
Total length (cm)	35.40 ± 2.34	35.51 ± 2.02	0.9028
Standard length (cm)	29.30 ± 2.06	28.92 ± 1.56	0.6156
Head length (cm)	9.12 ± 0.69	8.81 ± 0.60	0.2419
Back height (cm)	12.75 ± 1.01	12.28 ± 0.91	0.2361
Back width (cm)	5.75 ± 0.44	6.06 ± 0.43	0.0872
Carcass weight (g)	508.08 ± 119.06	503.92 ± 85.67	0.9216
Head weight (g)	240.54 ± 56.78	227.50 ± 33.84	0.4973

Each fish was considered an experimental unit (*n* = 12 individuals per variety). Results are presented as mean ± standard error. ^a,b^ Different letters indicate significant differences by Student’s *t*-test (*P* < 0.05).

Table 4 shows the impact of skin color phenotype on fillet quality parameters. Red tilapia had a higher pH value 2 h post-slaughter (*P* = 0.0362), lower L* value (*P* = 0.0060), lower b* value (*P* = 0.0468), lower hue angle (*P* = 0.0003), lower ThL (*P* = 0.0131), lower CL (*P* = 0.0140), lower hardness (*P* = 0.0019), higher cohesiveness (*P* = 0.0353), lower adhesiveness, and lower chewability (*P* = 0.0077) than black tilapia. There were no significant differences in fillet elasticity between varieties (*P* = 0.6587).

**Table 4:** Quality indicators of black and red Nile tilapia (*Oreochromis niloticus*) fillets.

Indicator	Variety		*P*-value
	Black	Red	
pH 2 h post slaughter	5.90^b^ ± 0.12	5.95^a^ ± 0.08	0.0362
L*	48.92^a^ ± 3.51	46.66^b^ ± 3.39	0.0060
a*	3.47 ± 1.72	4.23 ± 2.50	0.1284
b*	3.31^a^ ± 1.30	2.68^b^ ± 1.40	0.0468
Chroma	4.42 ± 1.83	4.17 ± 1.74	0.5654
Hue angle	0.71^a^ ± 0.24	0.45^b^ ± 0.34	0.0003
Water activity	0.99 ± 0.002	0.99 ± 0.002	0.6486
Thawing loss	9.65^a^ ± 0.56	7.85^b^ ± 0.15	0.0131
Cooking loss	20.32^a^ ± 0.56	18.30^b^ ± 0.28	0.0140
pH at 12 h after thawing	5.79 ± 0.15	5.78 ± 0.11	0.7686
Hardness (N)	75.27^a^ ± 4.06	51.51^b^ ± 4.63	0.0019
Cohesiveness	0.85^b^ ± 0.02	0.87^a^ ± 0.01	0.0353
Elasticity (mm)	1.00 ± 0.01	1.00 ± 0.01	0.6587
Adhesiveness	–0.28^a^ ± 0.04	–0.18^b^ ± 0.06	0.0104
Chewability (N/mm)	72.86^a^ ± 4.30	44.68^b^ ± 5.66	0.0077

Each fish was considered an experimental unit (*n* = 12 individuals per variety). Results are presented as mean ± standard error. L*, luminosity; a*, red-green axis; b*, yellow-blue axis ^a,b^ Different letters indicate significant differences by Student’s *t*-test (*P* < 0.05).

Regarding antioxidant capacity and oxidative metabolites, it was found that black tilapia had a higher content of conjugated dienes (*P* = 0.0420), lower DPPH^•^ scavenging activity (*P* = 0.0341), and lower ABTS^•+^ scavenging activity (*P* = 0.0424) (Table 5). There were no significant differences in muscle TBARS content (*P* = 0.5751) or carbonyl protein content (*P* = 0.8391) between black and red tilapia (Table 5).

**Table 5:** Antioxidant capacity (DPPH^•^ and ABTS^•+^) and oxidative metabolites in muscle tissues of black and red Nile tilapia.

Parameter	Variety		*P*-value
	Black	Red	
TBARS (nmol TBARS/mg protein)	0.93 ± 0.18	0.82 ± 0.09	0.5751
Conjugated dienes (μmol/g sample)	1.73^a^ ± 0.08	1.45^b^ ± 0.09	0.0420
Carbonylated protein (nmol carbonylated protein/mg protein)	2.61 ± 0.60	2.47 ± 0.27	0.8391
DPPH^•^ scavenging activity (%)	28.03^b^ ± 1.05	34.36^a^ ± 0.80	0.0341
ABTS^•+^ reducing activity (%)	25.92^b^ ± 0.90	29.10^a^ ± 0.46	0.0424

Each fish was considered an experimental unit (*n* = 7 individuals per variety). Results are presented as mean ± standard error. TBARS, thiobarbituric acid reactive substances; DPPH^•^, 2,2-diphenyl-1-picrylhydrazyl radical; ABTS^•+^, 2,2′-azino-bis(3-ethylbenzothiazoline-6-sulfonate radical. ^a,b^ Different letters indicate significant differences by Student’s *t*-test (*P* < 0.05).

**Fig 3:**
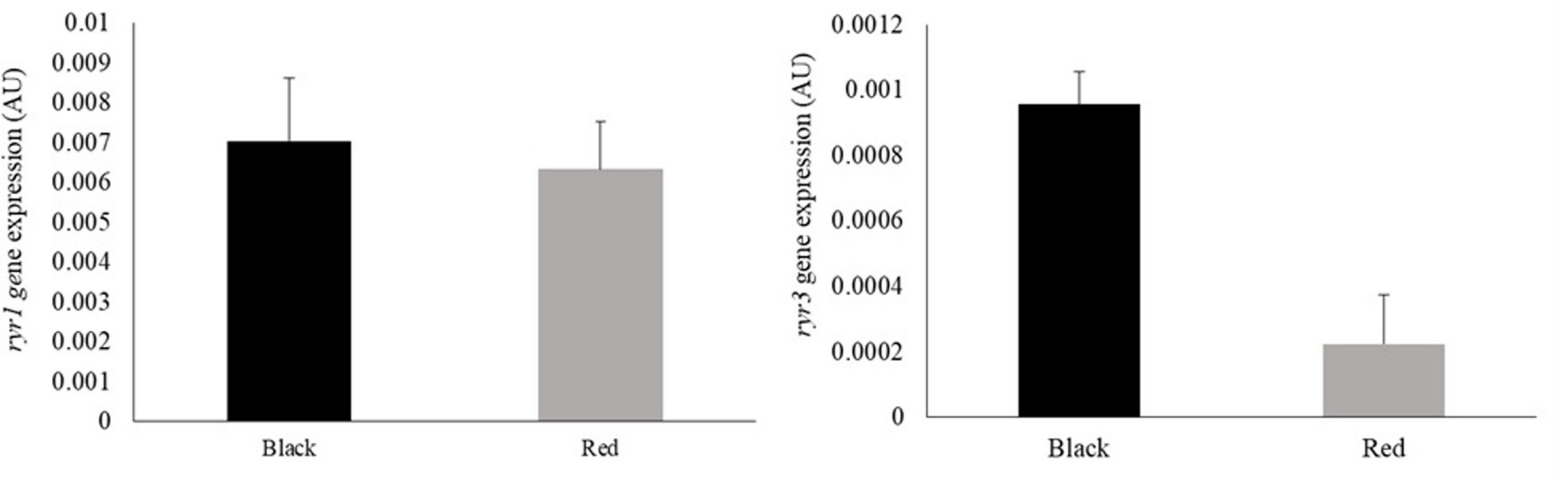
Expression of genes encoding ryanodine receptors 1 (*ryr1*) and 3 (*ryr3*) in the muscle of black and red Nile tilapia (*Oreochromis niloticus*). Each fish was considered an experimental unit (*n* = 7 individuals per variety). Results are expressed as arbitrary units (AU) and presented as mean ± standard error.

No significant differences were observed in the expression of *ryr1* (*P* = 0.7270) or *ryr3* (*P* = 0.2915) in the muscle of black and red tilapia (Fig 3).

The results of the skin resistance test are shown in Table 6. In the tensile and elongation test, skin color phenotype influenced tensile force (*P* = 0.0079), tensile strength (*P* = 0.0365), deformation (*P* = 0.0077), and elongation (*P* = 0.0004). The skin of red tilapia exhibited lower tensile force, tensile strength, deformation, and elongation than that of black tilapia. Furthermore, in the progressive tear test, tearing force (*P* = 0.0237) was influenced by tilapia variety, with red tilapia exhibiting lower tearing force than black tilapia. No significant effect was observed on tear strength (*P* = 0.01039).

**Table 6:** Skin resistance parameters of black and red Nile tilapia (*Oreochromis niloticus*).

Test	Parameter	Variety		*P*-value
		Black	Red	
Tensile and elongation test	Tensile force (N)	113.33^a^ ± 7.63	85.42^b^ ± 4.60	0.0079
	Tensile strength (N/mm)	13.15^a^ ± 0.62	10.81^b^ ± 0.73	0.0365
	Deformation (mm)	50.16^a^ ± 3.56	38.85^b^ ± 1.07	0.0077
	Elongation (%)	86.83^a^ ± 4.30	64.85^b^ ± 1.68	0.0004
Progressive tear test	Tearing force (N)	44.50^a^ ± 3.25	36.00^b^ ± 1.15	0.0237
	Tear strength (N/mm)	51.45 ± 3.02	45.70 ± 1.46	0.1039

Each fish was considered an experimental unit (*n* = 12 individuals per variety). Results are presented as mean ± standard error. ^a,b^ Different letters indicate significant differences by Student’s *t*-test (*P* < 0.05).

**Fig 4:**
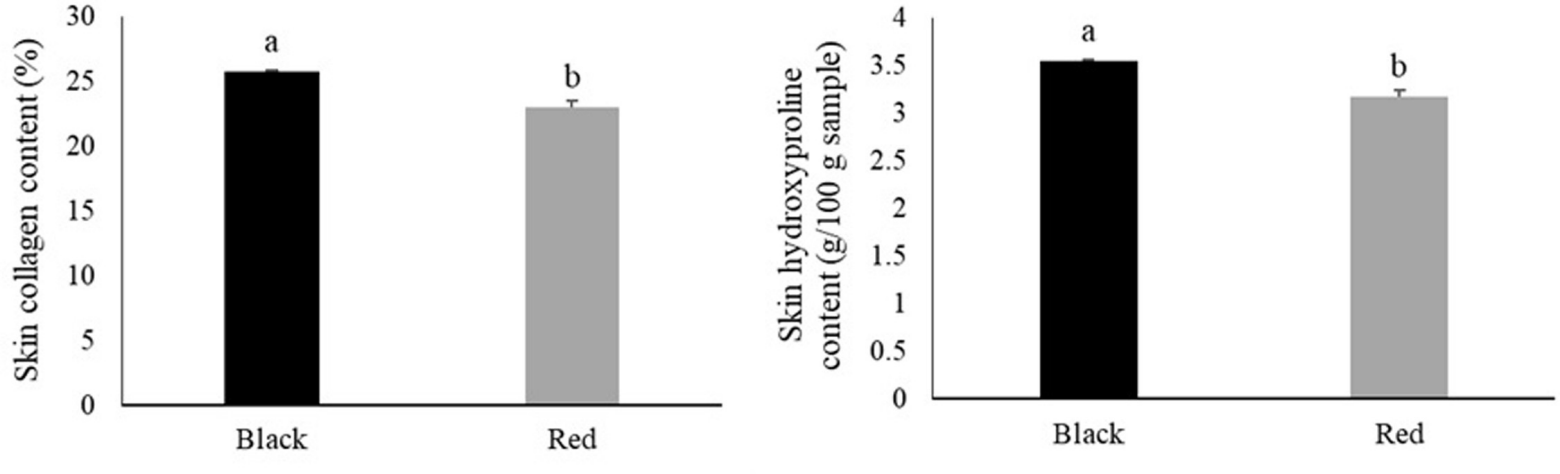
Hydroxyproline and collagen content of the skin of black and red Nile tilapia (*Oreochromis niloticus*). Each fish was considered an experimental unit (*n* = 7 individuals per variety). Results are presented as mean ± standard error. ^a,b^ Different letters indicate significant differences by Student’s *t*-test (*P* < 0.05).

Fig 4 shows the hydroxyproline and collagen contents of the skin of black and red tilapia. Skin color significantly influenced hydroxyproline (*P* = 0.0003) and collagen (*P* = 0.0003) contents. The highest contents were observed in the skin of black tilapia.

**Fig 5:**
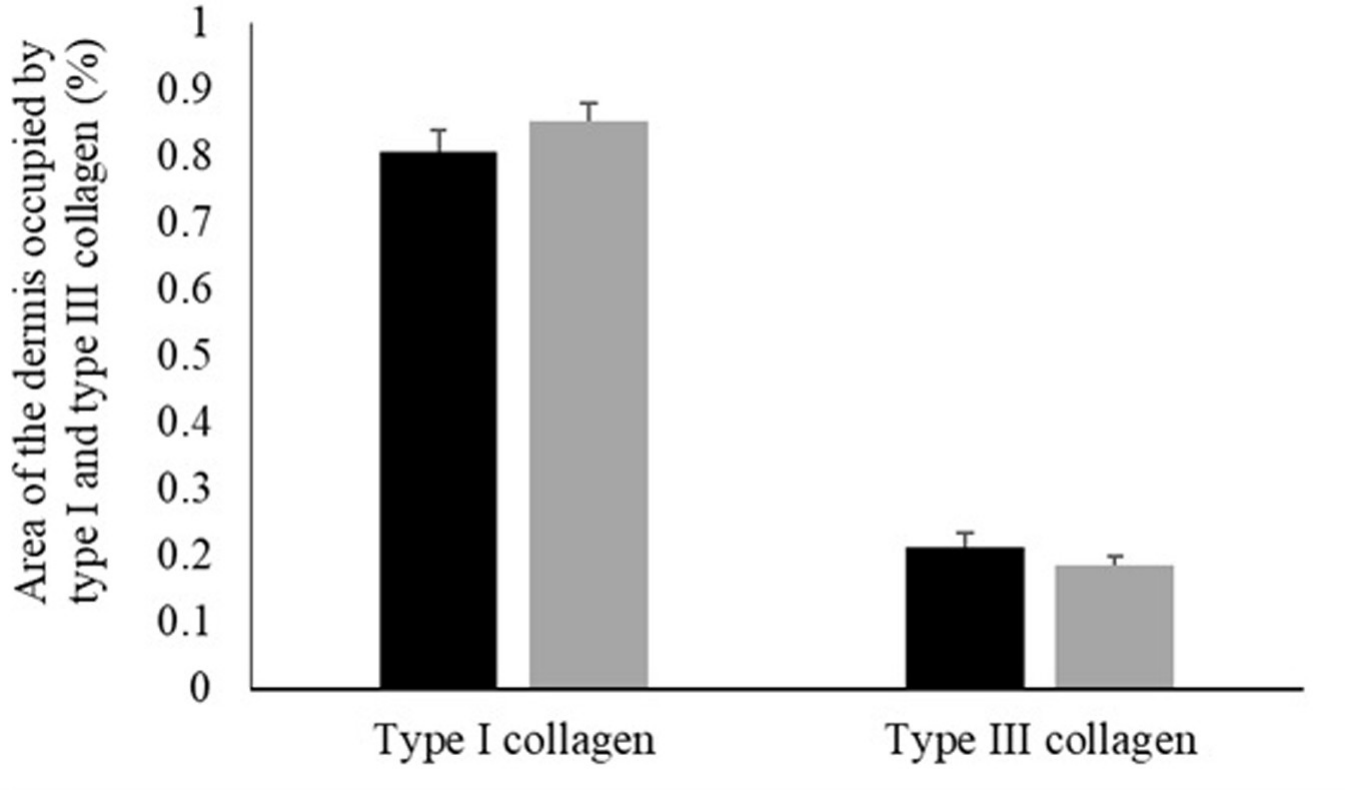
Determination of the area occupied by type I and type III collagen in the skin of black and red Nile tilapia (*Oreochromis niloticus*). Each fish was considered an experimental unit (*n* = 7 individuals per variety). Results are presented as mean ± standard error.

Fig 5 shows the area of skin occupied by type I and type III collagen. There was no significant effect of fish variety on the area occupied by type I (*P* = 0.2801) or type III (*P* = 0.2763) collagen.

**Fig 6:**
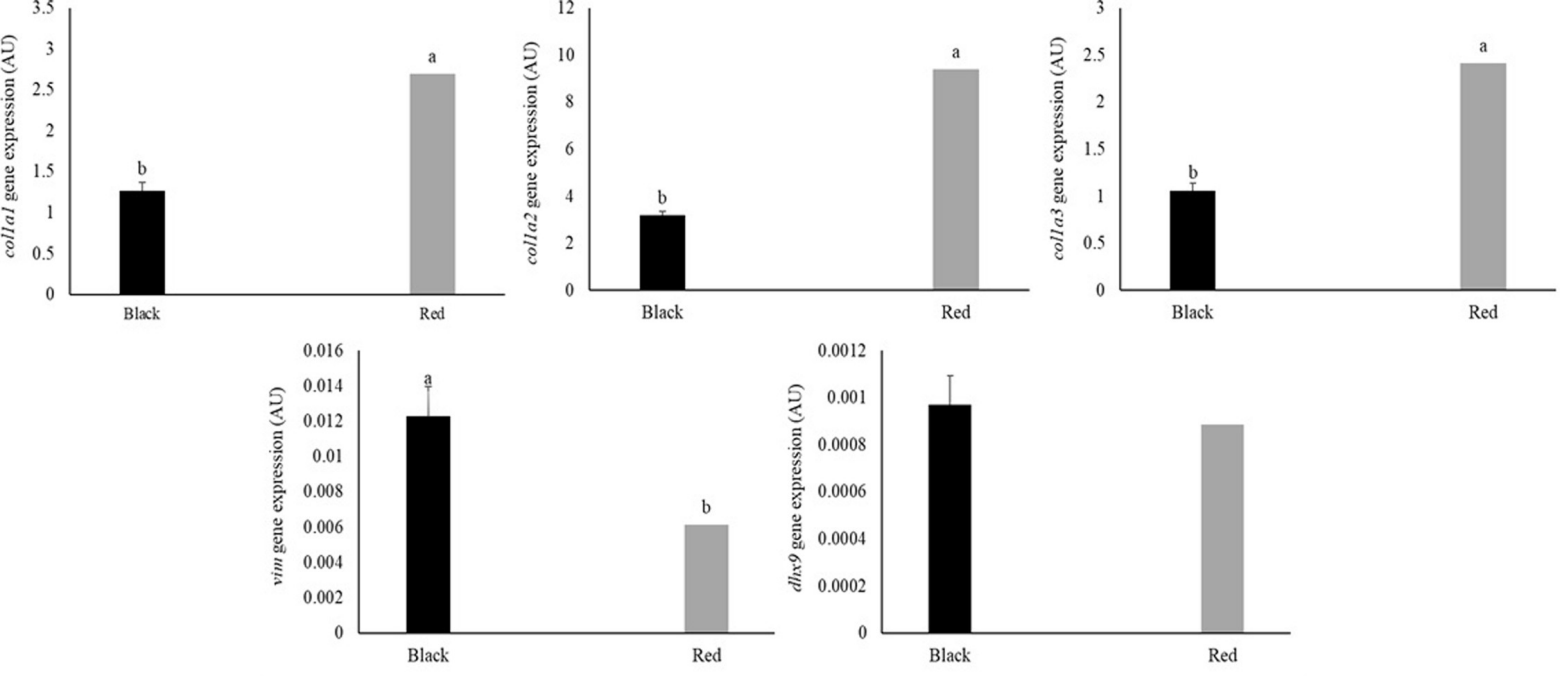
Expression of genes encoding collagen type I alpha 1 (*col1a1*), *2* (*col1a2*), and *3* (*col1a3*), vimentin (*vim*), and DEAH box helicase 9 (*dhx9*) in the skin of black and red Nile tilapia (*Oreochromis niloticus*). Each fish was considered an experimental unit (*n* = 7 individuals per variety). Results are expressed as arbitrary units (AU) and are presented as mean ± standard error. ^a,b^ Different letters indicate significant differences by Student’s *t*-test (*P* < 0.05).

Red tilapia exhibited a higher expression of *col1a1* (*P* = 0.02000), *col1a2* (*P* = 0.0032), and *col1a3* (*P* = 0.0141) in skin tissues than black tilapia (Fig 6). Regarding genes regulating collagen expression, *vim* expression was highest in black tilapia (*P* = 0.0067). The expression of the *dhx9* gene, which encodes ATP-dependent RNA helicase A, did not differ between tilapia varieties (*P* = 0.9259) (Fig 6).

**Fig 7:**
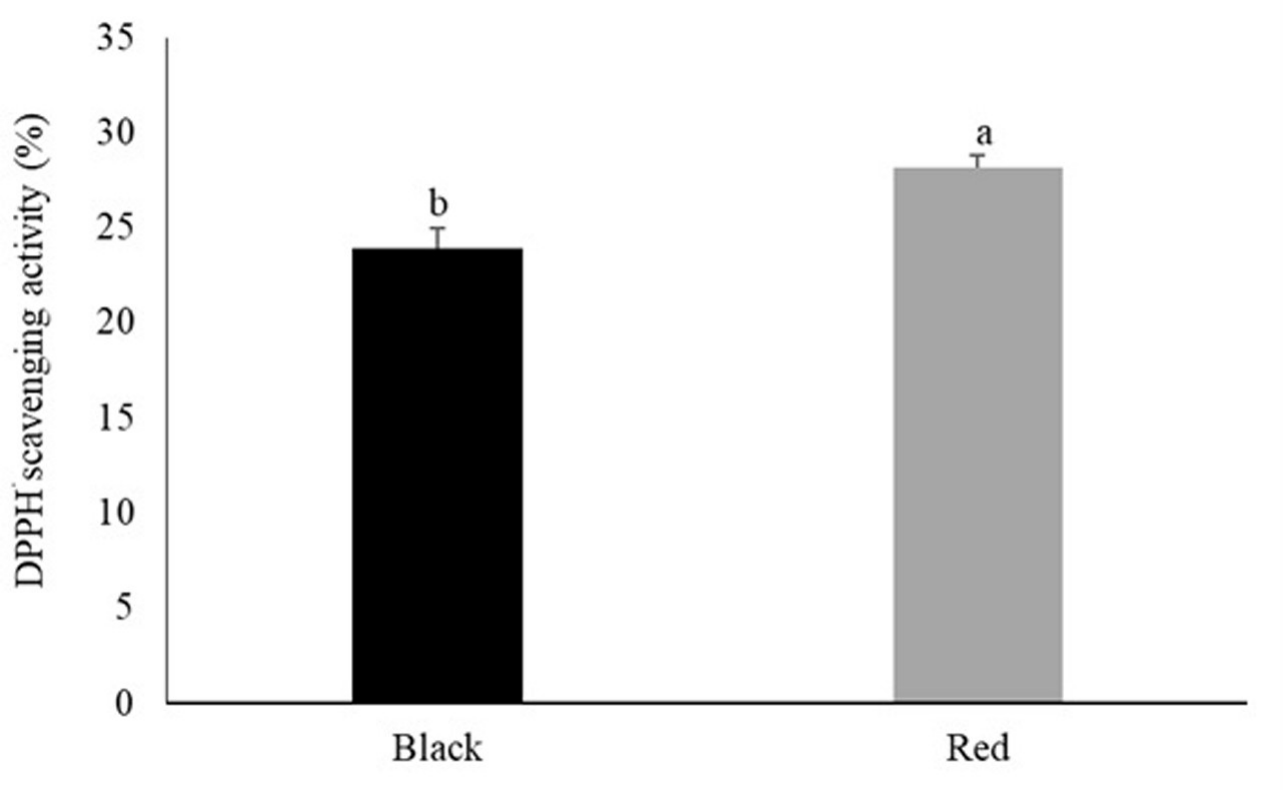
2,2-Diphenyl-1-picrylhydrazyl radical (DPPH^•^) scavenging activity of the skin of black and red Nile tilapia (*Oreochromis niloticus*). Each fish was considered an experimental unit (*n* = 7 individuals per variety). Results are presented as mean ± standard error. ^a,b^ Different letters indicate significant differences by Student’s *t*-test (*P* < 0.05).

Fig 7 shows the results of the antioxidant capacity of the skin. There was a significant difference (*P* = 0.0171) between varieties, with red tilapia exhibiting a higher antioxidant capacity than black tilapia.

## Discussion

This study aimed to biometrically characterize Nile tilapia of the GIFT strain exhibiting black or red skin color phenotypes and determine the effect of skin color on fillet and skin characteristics. No significant differences were observed between tilapia varieties in any of the biometric characteristics evaluated. The fish used in this study belong to the same lineage and therefore share the same genetic makeup. Although phenotypic variation in skin color was observed, biometric standardization eliminates the need for separating fish into different batches within the production system.

Fillet is the primary form in which tilapia meat is marketed, and it is regarded as a source of high-quality protein. Although no differences in fillet weight were observed between black and red tilapia, some fillet quality parameters were influenced by skin color phenotype. Tilapia with black skin exhibited a lower fillet pH value, higher L* value, higher b* value, and higher hue angle. Soares and Gonçalves [35] reported a similar pH value to that observed here. According to the authors, the pH is low compared with that observed in other studies, which suggested that the ideal pH of fish fillets is close to 7 [36]. The post-mortem decrease in pH is associated with the transformation of glycogen into lactic acid. Lactic accumulation leads to a decrease in fillet pH. Thus, fillet pH seems to be related to diet [37] and the genetic lineage of fish.

Post-mortem pH is an important indicator of meat quality and is directly related to the reduction in the muscle’s ability to retain water. A reduction in pH leads to the denaturation of muscle proteins and loss of solubility, affecting the texture and color of meat [38]. This effect was evidenced by the increase in ThL and CL in black tilapia fillets. Goes et al. [39] suggested a relationship between the expression of *ryr1* and *ryr3* genes during pre-slaughter stress and the water holding capacity of Nile tilapia fillets. The authors explained that the reduction in water retention in fillets from stressed fish may be due to a reduction in the expression of *ryr1*. The protein controls the liberation of Ca^2+^ from intracellular stores, leading to excess production of Ca^2+^ in the cytosol. These effects result in hypermetabolism and muscle contraction, thereby reducing post-mortem pH and causing protein denaturation and water loss in meat [40]. However, in our study, we did not observe a significant effect of fish variety on *ryr1* or *ryr3* expression. This finding suggests that possibly in conditions where fish are not in a state of stress there are other unknown metabolic mechanisms involved in the reduction of post-mortem pH.

**Fig 8:**
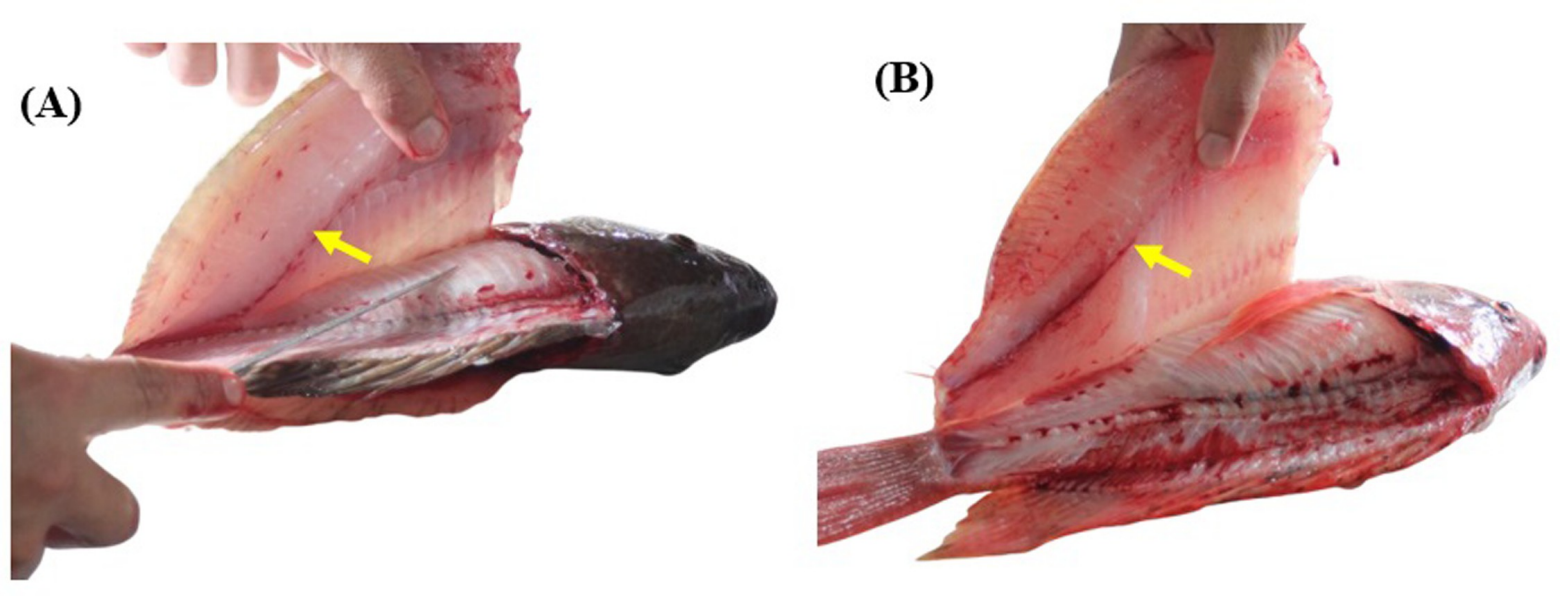
In vivo staining of fresh fillets of (A) black and (B) red Nile tilapia (*Oreochromis niloticus*). Source: Personal archive. The yellow arrow indicates the lateral line.

Meat color is an important criterion influencing consumer choice. Color is a qualitative attribute that indicates the physicochemical state of muscle fibers and is directly related to the amount of liquid and myoglobin present in muscles [41,42]. Here, it was observed that fillets of black and red tilapia differed in color parameters. The L* and b* of black tilapia fillets indicated a tendency toward reflection of white, resulting in a lighter color. Additionally, the hue angle, which refers to the perceived color of fillets, was very close to 0°, indicating a reddish color. The color components of fillets are dominated by the pigment myoglobin [43]. In black tilapia, fillets are mainly composed of light-colored muscle, also containing dark-colored muscle, particularly along the lateral line (Fig 8). This dark muscle contains large amounts of myoglobin [44]. In red tilapia, fillets are composed mainly of red-colored muscle, as well as dark muscle along the lateral line (Fig 8).

In this tilapia variety, although the hue angle was lower than that of black tilapia, it was close to 0°, indicating a reddish color. No previous study had evaluated the color parameters of red tilapia fillets, and, to date, no research has investigated whether the same genetic factors that control skin color are responsible for fillet color. The color differences between fillets from black and red tilapia seem to be mainly related to the location and quantity of myoglobin.

According to Purslow et al. [43], the color of myoglobin may change according to its biochemical state and degree of oxidation or reduction. In addition to being the main pigment in meat, myoglobin acts as a pro-oxidant factor, initiating the process of lipid oxidation. As reviewed by Domínguez et al. [45] lipid oxidation is the main non-microbial cause of deterioration in meat quality. The presence of polyunsaturated fatty acids (PUFAs) and heme pigments makes fish meat more prone to lipid oxidation than other meats, given that these fatty acids are easily oxidized [46].

The presence of metals, in free form or as heme proteins, is one of the main factors determining the oxidative stability of meat. Metals such as iron and copper act as catalysts for different processes and stages of lipid oxidation [45,47]. According to Domínguez et al. [45], most of the iron in biological tissues occurs in the form of hemoglobin and myoglobin, the most abundant heme proteins in meat. Oxidation of heme proteins negatively affects meat color, thereby influencing consumer acceptability. Previous study demonstrated that the oxidation of lipids and heme proteins in meat occurs simultaneously, and these processes seem to stimulate each other [48].

For example, oxyheme oxidation results in the formation of Met–heme and hydroperoxyl or superoxide radicals, which are converted into hydrogen peroxide [45]. Met–heme can react with hydrogen peroxide or pre-formed lipid hydroperoxide to produce highly reactive ferryl heme radicals [49]. All reactive oxygen species generated during oxyheme oxidation, as well as ferryl radicals, can abstract hydrogen from PUFAs and initiate lipid oxidation [48]. Thus, the oxidative stability and color of meat depend on the balance between anti- and pro-oxidant compounds [46].

The oxidative stability of fillets was assessed by determining parameters of primary (conjugated dienes) and secondary (TBARS) lipid oxidation and the antioxidant capacity of tilapia muscles (DPPH^•^ and ABTS^•+^). Black tilapia fillets exhibited lower oxidative stability, given the greater primary lipid oxidation and lower antioxidant capacity. Thus, black tilapia fillets have lower oxidative and color stability. The lighter color of black tilapia fillets may be a reflection of the increased production of primary lipid oxidation products. It was expected that the conversion of myoglobin to metmyoglobin would change fillet color from bright red to brown. However, a brown color was not observed, possibly due to the lower amount of myoglobin in black tilapia fillets, which are mostly composed of light muscle [50].

In summary, the lower oxidative and color stability of black tilapia fillets might be related to their lower pH value. Several conditions can initiate the oxidation cycle of heme proteins and lipids [51]. For example, in meat with low pH values (<7), the solubility of iron is increased, favoring heme protein oxidation [51]. Furthermore, the findings may be related to the lipid content of black tilapia muscles. Garduño-Lugo et al. [52] demonstrated that the fillet lipid content of wild-type Nile tilapia was higher than that of a red tilapia hybrid (2.07% vs. 0.33%, respectively).

Oxidative reactions are known to reduce the nutritional value of meat, resulting from a loss of essential fatty acids and vitamins [45]. Furthermore, such reactions affect sensory quality by altering the color, texture, and consistency of meat, thereby influencing consumer acceptance [53]. Here, the texture profile of fillets was assessed to gain insight into their quality and potential acceptance by consumers. The analysis evaluates attributes that influence the perception of meat quality during chewing [26]. The results demonstrated the negative effects of primary oxidation on the texture profile of fillets. Black tilapia samples had greater hardness, stickiness, and chewiness, as well as lower cohesiveness. Thus, these fillets had greater resistance to deformation, requiring more force to be chewed but falling apart more easily because of their reduced capacity to maintain structural integrity. As such, there is a greater tendency to adhere to surfaces (utensils, teeth, and lips). These effects can be attributed to lipid oxidation, as lipids are crucial for maintaining moisture (juiciness) and tenderness [54]. Together, these characteristics indicate that black tilapia fillets are less appealing than red tilapia fillets.

As stated by Domínguez et al. [45], deterioration of the qualitative attributes of fillets begins when fish are slaughtered and continues progressively until the final product is consumed. Therefore, it is necessary to adopt strategies to increase the antioxidant capacity of black tilapia fillets. Furthermore, the handling, processing, and storage of fillets must be strictly controlled to avoid the progression of these undesirable reactions and minimize economic losses [55].

Fish meat is mainly sold in the form of fillet cuts. Such processing generates large amounts of waste, including viscera, head, scales, and skin. These materials could be repurposed, minimizing the environmental impacts caused by the indiscriminate disposal of fish waste [6]. For this, it is essential to understand the parameters related to skin resistance. Oliveira et al. [56], in evaluating the quality parameters of tilapia skin, observed that fish leather had a tensile strength of 13.52 N/mm^−2^ and a tear strength of 53.85 N/mm^−1^. These values are similar to those observed in the present study for black tilapia (13.15 N/mm^−2^ and 51.45 N/mm^−1^, respectively). Although red tilapia exhibited lower tensile (10.81 N/mm^−2^) and tear (45.70 N/mm^−1^) strengths than black tilapia, these values are still higher than those observed for some mammals, such as rabbits (8.98 N/mm^−2^ and 24.25 N/mm^−1^, respectively) [56]. Such findings confirm the quality of tilapia leather and its potential use in the manufacture of clothes and accessories.

Tilapia skin has also been applied in biomedicine for the treatment of burns and extraction of bioactive compounds, such as collagen. This fibrous insoluble protein is present in large amounts in tilapia skin [10,11,13]. The collagen yield of red tilapia was lower than that of black tilapia (23.01% vs. 25.76%, respectively); nevertheless, these values are higher than those previously reported for red tilapia strains (22.58% [57] and 19.04% [16]). Medina-Medrano et al. [57] assessed the antioxidant properties of collagen from red tilapia skin and concluded that it shows potential as an active ingredient of nutraceuticals, pharmaceuticals, and functional foods. The results of the current study are in agreement with these observations, as the antioxidant capacity of red tilapia skin was higher than that of black tilapia skin.

Hydroxyproline, an amino acid that composes type I collagen, was observed at higher concentrations in the skin of black tilapia. This result is associated with the higher collagen content of this tilapia variety, contrary to the findings of Reátegui-Pinedo et al. [16]. The authors found no significant differences in the skin hydroxyproline content between different tilapia strains, including the red variety.

Type I collagen is composed of two types of peptides, α1 and α2, which fold into a triple helix at a 2:1 ratio, that is, two α1 chains for every one α2 chain. The translation and folding rates of collagen peptides must be tightly regulated to avoid structural imbalances and the synthesis of α1 homotrimers. DEAH box helicase 9 and vimentin play important roles in processes related to structural RNA changes, directly binding to the post-transcriptional element and promoting its unwinding, thereby increasing translation efficiency [58].

The expression of type I collagen subunits α1, α2, and α3 was increased in the skin of red tilapia. However, the expression of genes encoding proteins that regulate the translation and folding of collagen peptides (*vim* and *dhx9*) was higher in black tilapia. This result suggests that, although black tilapia has a lower gene expression of type I collagen subunits, its collagen synthesis may be more efficient. Genes encoding proteins that are crucial for the translation and stability of collagen mRNA are more highly expressed in the skin of these individuals.

In this study, skin color phenotype did not influence the biometric characteristics of GIFT Nile tilapia. However, tilapia varieties differed with regard to meat quality and skin texture. Red tilapia fillets had superior qualitative attributes, aspects that may greatly influence consumer acceptance. For instance, fillet pH was higher and b*, L*, ThL, and CL were lower in red tilapia fillets than in black tilapia fillets. Regarding skin quality parameters, black tilapia tended to have tougher skin than red tilapia. There was also a significant difference in collagen yield and the gene expression of type I collagen subunits and cofactors, indicating greater efficiency in the synthesis of type I collagen in the skin of black tilapia.

## Conclusions

The results suggest that the skin color phenotype of Nile tilapia may be associated with important metabolic pathways influencing fillet and skin characteristics. These findings can support future research aimed at identifying optimal uses for Nile tilapia varieties and optimizing the utilization of waste.
